# Red Blood Cell Distribution Width as a Novel Parameter in Canine Disorders: Literature Review and Future Prospective

**DOI:** 10.3390/ani13060985

**Published:** 2023-03-08

**Authors:** Arianna Miglio, Carlotta Valente, Carlo Guglielmini

**Affiliations:** 1 Department of Veterinary Medicine, University of Perugia, Via San Costanzo 4, 06126 Perugia, Italy; arianna.miglio@unipg.it; 2 Department of Animal Medicine, Production and Health, University of Padua, Viale dell’Università 16, 35020 Legnaro, Italy; carlotta.valente@unipd.it

**Keywords:** dog, RDW, biomarker, hematology, prognosis, mortality

## Abstract

**Simple Summary:**

Red blood cell distribution width (RDW) is a laboratory parameter that is automatically calculated by most hematology analyzers and reflects the degree of variation in the erythrocyte size. Various studies have shown that RDW can be used as a diagnostic and prognostic biomarker in many hematological and non-hematological disorders in humans. This narrative review aimed to summarize the findings of available studies investigating the relationship between RDW and various canine disorders.

**Abstract:**

Red blood cell distribution width (RDW) is a quantitative laboratory parameter applied for the measurement of anisocytosis and is a reliable and inexpensive method for clinical classification of anemia. An increased RDW reflects a great heterogeneity in the size of red blood cells typical of regenerative and iron-deficiency anemia. In humans, numerous and recent studies have shown a relationship between increased RDW and the risk of morbidity and mortality in patients with various disorders. In particular, a strong association has been established between changes in RDW and the risk of adverse outcome in humans with diseases affecting different organs or systems. Following the human literature, some studies have recently been conducted trying to clarify the clinical role of RDW in various animal disorders, particularly in dogs. In this review, we summarize and critically evaluate the results of the studies based on the measurement of RDW in dogs. We also emphasize the need for further and more extensive studies on the use of this simple and inexpensive parameter in animals.

## 1. Introduction

**Figure 1:**
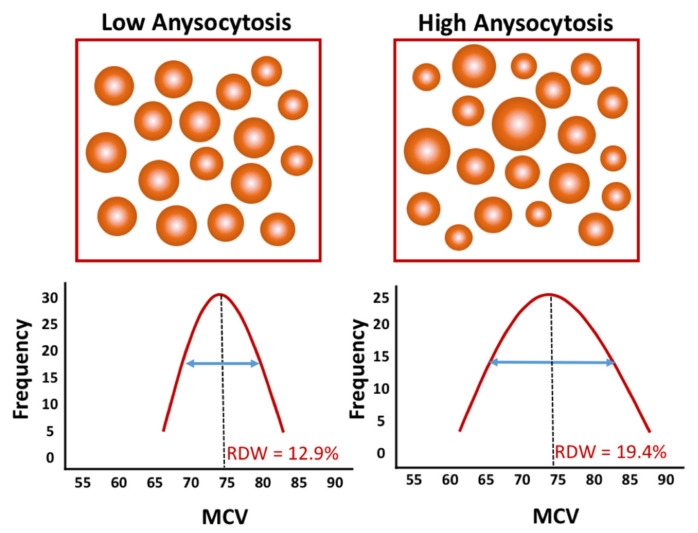
Relationship between erythrocyte volume (i.e., mean corpuscular volume, MCV) and red blood cell distribution width (RDW) in two dogs with the same MCV (i.e., 74.8 fL) but different degrees of anisocytosis.

Red blood cell distribution width (RDW) is a simple and inexpensive erythrocyte index commonly included in routine blood test reflecting the degree of variation in the erythrocyte size, namely anisocytosis. Red blood cell distribution width can be influenced by a change in the number of large or small red blood cells (RBCs) [1,2,3]. Most hematology analyzers automatically calculate this parameter by dividing the standard deviation (SD) of erythrocyte volume by the mean corpuscular volume (MCV) and multiplying by a factor of 100 to express it as a percentage [1,2,3]. Thus, it is easily accessible in most medical institutions due to the low technical requirements. Red blood cell distribution width is commonly used in conjunction with other complete blood count (CBC) parameters such as MCV, hemoglobin (Hb), and hematocrit (HCT) for the differential diagnosis and classification of anemia (i.e., regenerative, non-regenerative, and iron-deficiency anemia) and bone marrow dysfunction [1,2]. However, RDW is more accurate than MCV for identifying anisocytosis, as it is highly dependent on the degree of SD of erythrocyte volume. The latter could be high (high anisocytosis) or low (low anisocytosis) even with the same value of mean volume of RBC population (Figure 1). Red blood cell distribution width is usually expressed as a coefficient of variation, the unit of which is %, of the erythrocyte size distribution [2]. An RDW value below the standard reference range is rare and clinically insignificant, while values above the reference range suggest the presence of anisocytosis. This finding is due to the presence of small and/or large RBCs, indicating impaired erythropoiesis, shortened RBC life span, or premature release of immature RBCs [3].

**Figure 2:**
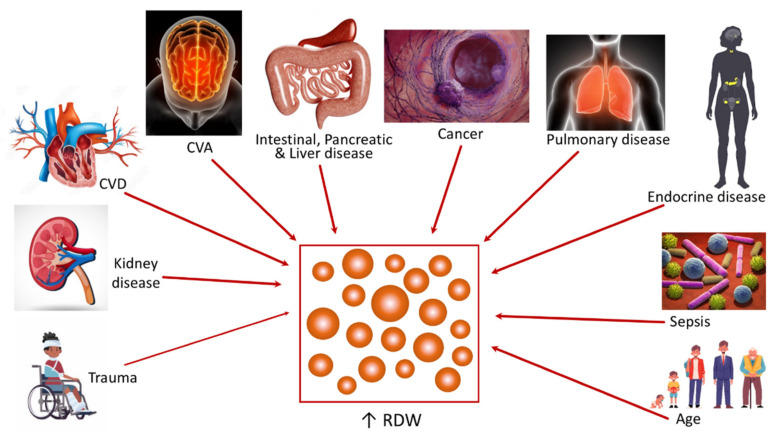
Schematic diagram representing different disorders characterized by increased red blood cell distribution width (RDW) in human patients. CVA: cerebrovascular accident; CVD: cardiovascular disease; IBD: inflammatory bowel disease.

Recently, the evaluation of RDW has gained considerable importance in humans, due to its multiple clinical applications. Various studies have shown that anisocytosis could be related to various disorders such as cardiovascular disease, infections, cancer, endocrine disease, pulmonary disease, gastrointestinal and liver disease, kidney disease, sepsis, neurological disorders, and fracture, as well as other acute or chronic conditions [4,5,6]. In addition to its value for diagnosing a variety of disorders, increased RDW has been shown to play an important role for prognosis in several clinical settings. Thus, the value of RDW is now considered to be an independent and powerful risk factor for an adverse outcome in the general population [2,3,4,5,6,7,8,9] (Figure 2).

Similar to the developments in human medicine, some studies investigating the use of RDW as a novel marker have also been recently published in various animal clinical settings. In particular, these studies have been conducted in small animals with cardiovascular disease [10,11,12,13,14,15] and other various disorders [16,17,18,19,20,21,22]. Therefore, the aim of this review is to provide the most relevant information on RDW evaluation in the dog. Possible pathophysiological explanations of the correlation between anisocytosis and the underlying disorder will be discussed. Finally, we will try to give some insights on the future prospective use of RDW as an independent diagnostic, as well as prognostic, parameter in different canine diseases.

## 2. Red Blood Cell Distribution Width, Hematological Analyzers, and Patient Characteristics

The methods used for erythrocytes analysis and RDW calculation differ widely among the most commonly used hematological analyzers, so that there is still a high degree of technological heterogeneity among them [2,23,24,25]. Human studies have shown that RDW values obtained using different hematological analyzers are not equivalent. This lack of harmonization limits the comparability of RDW values obtained using different hematological analyzers and hinders the use of identical reference ranges and decision thresholds across different clinical investigations [2,23]. In the dog, the laser-based hematology system is considered the gold standard for determining the CBC, but RDW values obtained using different hematological analyzers may vary by manufacturer, laboratory, and population tested [16,24,25,26].

Physiological variations of RDW associated with patient characteristics have been reported in humans. In particular, RDW can vary with age [27], sex [28,29], physical exercise [30], and pregnancy [31], although the relationship between anisocytosis and gender appears contradictory across different epidemiological investigations [2]. Breed-related but not age- and sex-related differences were reported in a clinically healthy dog [32], while age was positively, but weakly correlated with RDW in dogs with mitral valve disease (MVD) due to myxomatous degeneration [13] and pulmonary hypertension (PH) [11].

## 3. Red Blood Cell Distribution Width and Cardiovascular Disease

### 3.1. RDW in Mitral Valve Disease

Red blood cell distribution width has been studied in many human cardiovascular disorders and has been shown to be a useful predictor of adverse outcome in patients with acute and chronic heart failure (HF) [2,33].

Two studies [10,13] specifically evaluated RDW in dogs with MVD, which is the most common cardiac disease resulting in HF in the dog. In the first observational study, Guglielmini et al. [10], retrospectively included 27 clinically healthy dogs and 135 dogs with MVD with compensated (87/135) and decompensated HF (48/135). No significant difference was found between groups, suggesting the absence of an association between RDW and MVD or between dogs with compensated and decompensated HF. Differences in etiology and pathophysiology of HF between humans and dogs have been hypothesized to cause the failure of RDW elevation in dogs with HF [10]. Conversely, a more recent study from the same group [13] investigated the prognostic role of RDW in 146 dogs with MVD at various stages of the disease using a multivariable regression analysis. The results indicated that increase in RDW was significantly associated with a negative outcome in these animals. Specifically, per each 1% increase of RDW, there was an approximately 20% increased risk of all-cause mortality at six months, and a cut off value of RDW >13.6% was associated with a shorter survival time in the dogs studied [13]. Thus, RDW seems to play a useful prognostic role but a less important diagnostic role in dogs with MVD.

### 3.2. RDW in Pulmonary Arterial Hypertension and Heartworm Disease

Pulmonary hypertension refers to an increase in pulmonary artery pressure due to various cardiovascular, respiratory, and systemic diseases [34]. Pulmonary hypertension can be classified according to the underlying pathophysiological mechanism in precapillary PH and post-capillary PH (i.e., PH associated with left-sided cardiac disease) [34]. In humans, RDW is a useful predictive biomarker of disease severity and negative outcome in patients with PH and pulmonary embolism [35,36].

The relationship between RDW and PH has been evaluated in two retrospective studies in dogs [11,12]. Furthermore, an additional study evaluated the RDW in dogs with heartworm disease (HWD) [17], which is a frequent cause of precapillary PH in this species [17,37]. Swann et al. [12], in a study including 44 dogs with PH and 79 control dogs, identified a significantly increased RDW in dogs with precapillary PH secondary to different causes compared to healthy dogs, but this increase was not observed in dogs with postcapillary PH. Moreover, there was no difference in RDW between dogs with mild and those with severe PH nor in median survival time between dogs with PH divided according to RDW values [12]. However, the evaluation of the prognostic role of RDW was limited by the small sample size and the low mortality rate.

Mazzotta et al. [11], in a study on 50 dogs with MVD but without PH, 32 dogs with PH secondary to MVD (i.e., postcapillary PH), and 26 with precapillary PH observed an increased RDW in dogs with precapillary and postcapillary PH compared to clinically healthy dogs. Underlying pathophysiologic processes associated with PH were considered responsible for increased RDW in these dogs [11].

A recent study [17] evaluated the correlation between RDW values in 86 dogs with HWD at various disease stages, and 16 of them had PH. Only these latter dogs with more severe HWD had significantly increased RDW and decreased HCT and Hb compared to control dogs. Thus, anemia and anisocytosis are common complications of severe HWD associated with PH. 

The combined results of these studies suggest that RDW may provide diagnostic information but is not a useful predictor of PH severity in the dog. Furthermore, the prognostic role of RDW in dogs with PH requires further studies.

## 4. Red Blood Cell Distribution Width and Pancreatitis

Acute pancreatitis (AP) is a severe and complex inflammatory disease with a high mortality rate. Therefore, the assessment of the severity of this disease at the time of admission is extremely useful for clinicians to improve treatment approach and reduce mortality [8,9,38,39,40,41]. In humans with AP, RDW and RDW to total calcium ratio (RDW/Ca) have been strongly associated with a poor outcome [42,43,44,45,46]. A recent multicenter retrospective study evaluated the prognostic value of RDW and RDW/Ca as well as other routine clinico-pathological parameters in 70 dogs with AP [16]. Red blood cell distribution width and RDW/Ca were significantly increased in 19 (28.1%) non-surviving dogs compared with 51 (71.9%) surviving dogs, and an RDW of >12.7% had good accuracy in predicting short-term death (i.e., dead within 14 days of admission). Using a multivariable regression analysis, RDW was an independent predictor of poor outcome, and dogs with RDW >12.7% had a fivefold risk of mortality compared to those with RDW ≤12.7%.

## 5. Red Blood Cell Distribution Width and Acute Trauma

A recent retrospective study evaluated the prognostic role of several hematological parameters, including RDW, in 129 dogs presenting within 24 h of acute traumatic injury. Of these dogs, 109 (84.5%) survived and 20 (15.5%) died or were euthanized during hospitalization. Red blood cell distribution width was not significantly different between survivors and non-survivors and was not an independent predictor of adverse outcome [22]. Conversely, several studies have demonstrated that RDW is predictive of mortality in traumatized human patients [29,47], and serial measurement of this parameter is recommended to predict 28-day mortality in those with suspected severe trauma [47].

## 6. Red Blood Cell Distribution Width and Critical Ill Dogs/Hospitalized Dogs/Dogs Admitted to Intensive Care Unit

Recent articles have reported the results of RDW evaluation in animals with different severe diseases [19,20,21]. Ludwik et al. [20] assessed the relationship between RDW and mortality using logistic regression modeling in 6661 hospitalized animals, including 5183 dogs and 1478 cats. This retrospective, single-center study included animals presented to emergency service and admitted to the intensive care unit (ICU) for medical disease (e.g., systemic, digestive, neurological, and myeloproliferative), surgical disease, or both medical and surgical disease. Animals were stratified into quartiles based on RDW at presentation. Results demonstrated that hospitalized dogs with higher RDW have greater odds of all-cause in-hospital mortality than those with lower RDW. A similar association between RDW and mortality was not found in cats [20]. Another retrospective study aimed to determine whether increased RDW at presentation was associated with in-hospital mortality and length of hospitalization in 127 critically ill dogs admitted to the ICU for heterogeneous disorders [19]. No association was found between RDW and in-hospital mortality or length of hospitalization in the studied dogs. A third prospective study investigated the accuracy of RDW at presentation to predict disease severity and mortality in 111 dogs consecutively presented to the ICU primarily because of gastrointestinal, neurologic, cardiac, respiratory, and urogenital disease [21]. In this heterogeneous population, 95 dogs (85.6%) and 16 (14.4%) survived to hospital discharge and did not survive to discharge, respectively. No significant difference in RDW was found between survivors and non-survivors at hospital discharge or 30 days after admission. 

Differences in study design and patient population with variable mortality rate are likely responsible for the discrepant results of these studies. However, interestingly, an association was found between increased RDW and poor outcome in the study that included the largest number of animals and used a more powerful statistical analysis [20]. The results of this study are similar to those observed in humans, which demonstrated the negative prognostic role of RDW in critically ill patients [48,49].

## 7. Red Blood Cell Distribution Width and Miscellaneous Diseases

Martinez et al. [18] retrospectively evaluated RDW in 79 clinically healthy dogs and 276 dogs of different breeds with several miscellaneous disorders, including endocrine, neurological, respiratory, hematological, cardiovascular, hepatic, and pancreatic diseases. Significantly increased RDW was found in dogs with immune-mediated hemolytic anemia and thrombocytopenia, hyperadrenocorticism, hypothyroidism, hepatic vascular anomaly, pneumonia, chronic kidney disease, multi-centric lymphoma, or MVD, but not in those with diabetes, meningitis, epilepsy, hepatitis, pancreatitis, inflammatory bowel disease, eosinophilic broncho-pneumopathy, or chronic bronchitis. The prognostic role of RDW was not evaluated, and the small number of animals affected by many of the diseases studied limits the robustness of the results obtained. 

**Figure 3:**
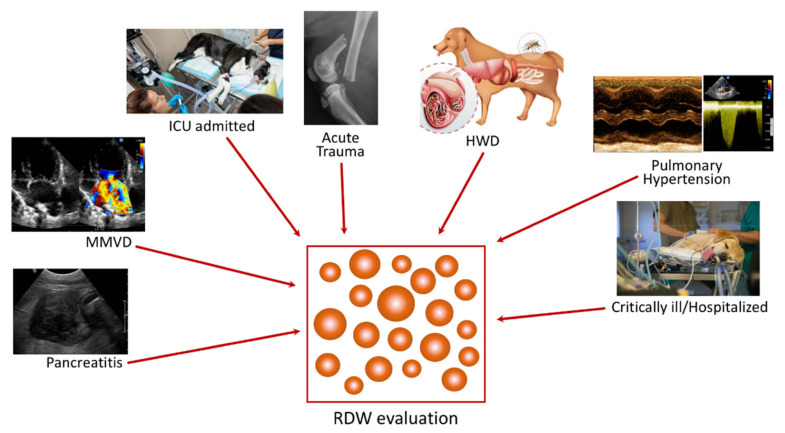
Schematic diagram representing different disorders in which red blood cell distribution width (RDW) has been evaluated in dogs. HWD: heartworm disease; ICU: intensive care unit; MMVD: myxomatous mitral valve disease.

Canine disorders in which RDW evaluation has been carried out and summary of studies exploring associations between RDW and these disorders are depicted and reported in Figure 3 and Table 1, respectively.

## 8. Pathophysiological Mechanisms of Increased RDW

The precise pathophysiological mechanisms underlying increases in RDW in various systemic disorders are not yet clear. The crucial question is whether anisocytosis is an independent risk factor for a specific disease or whether it represents a concomitant underlying metabolic and/or biological abnormality affecting RBCs [2,3]. In humans, several hypotheses have been proposed to explain this phenomenon, including anemia due to reduced erythropoietin synthesis and/or responsiveness, an underlying inflammatory state, oxidative stress, poor nutritional status, dyslipidemia, and the deformability or fragmentation properties of RBCs [2,3].

### 8.1. Anemia (Erythropoietin Synthesis and Responsiveness) and Iron Metabolism

Erythropoietin regulates the production and maturation of RBCs and is considered one of the main factors influencing RDW. Indeed, an increase in RDW reflects the presence of immature RBCs in the peripheral circulation, which could be due to reduced functional iron availability or inhibition of erythropoietin synthesis or activity. For example, the potential mechanisms underlying the development of anemia in humans with cardiovascular disease and HF appear to be multifactorial. These mechanisms include decreased erythropoietin production, ineffective erythropoiesis, iron deficiency and impaired iron metabolism, anemia from chronic disease and inflammatory state, malnutrition (nutritional deficiency of iron, vitamin B12, and folate), and comorbidities such as impaired renal function and cardio-renal anemia syndrome [50,51,52]. In dogs, the association between anemia and increased RDW were found only in those with PH of different causes, including HWD [11,17]. In contrast, no correlation was found between RDW and anemia in dogs with AP [16], and anemia is not associated with a poor prognosis in these animals [53,54,55]. Furthermore, some studies [10,56] have demonstrated a low prevalence of mild anemia in dogs with MVD and other cardiac diseases, and anemia associated with pre-renal azotemia was present only in animals with advanced HF. These findings suggest that the cardio-renal anemia syndrome described in humans is not a major problem in dogs with cardiac disease.

Red blood cell distribution width is influenced by iron metabolism, and impaired iron metabolism is characterized by the presence of higher RDW, low Hb, and low MCV [33]. The increased mobilization of stored iron is mediated by the overproduction of hepcidin (the main regulator of iron metabolism) in response to the inflammatory cytokine interleukin-6 (IL-6), which is strongly associated with increased RDW. Furthermore, IL-6 increases ferritin expression and iron retention within macrophages, which leads to decreased circulating iron level and limited iron availability to erythroid cells [57]. One study demonstrated that low serum/plasma iron but not RDW is associated with mortality in dogs with acute trauma [22], but the correlation between RDW and iron metabolism has not been investigated.

### 8.2. Inflammatory State

Several human studies suggest that an underlying inflammatory state can increase RDW by influencing erythropoiesis. In particular, it appears that pro-inflammatory cytokines, such as TNF-α, IL-1β, and IL-6, reduce renal erythropoietin (EPO) synthesis and desensitize erythroid progenitor cells to EPO by suppressing EPO gene expression and downregulating EPO receptor expression. These mechanisms induce blockage of erythroid progenitor cell proliferation, reduce RBC maturation and survival in the bone marrow, and cause larger and less mature RBCs (i.e., reticulocytes) to enter the peripheral blood, resulting in anisocytosis [30,50]. Numerous studies support this hypothesis by reporting the significant relationship between RDW and systemic inflammatory parameters such as white blood cells (WBC), neutrophil and lymphocyte counts, and C-reactive protein [22,41,44,50,58,59,60,61,62]. In contrast, only scant and sometimes conflicting data on this topic have been reported in the dog [11,13,16,18,19,20,22]. No correlation was found between the increase in RDW and WBC in dogs with compensated and decompensated MVD [10]. On the other hand, in dogs [11], as in humans [35,63] with PH, a significant association between WBC and RDW has been identified, suggesting a possible relationship between an inflammatory state and/or a stress response and anisocytosis in some types of PH. Furthermore, Guglielmini et al. [16] identified a positive correlation between RDW and WBC, neutrophil count, and neutrophil-to-lymphocyte ratio in dogs with AP, suggesting that the systemic inflammatory response is the underlying cause of anisocytosis. On the other hand, Garcia-Arce et al. [19] found no significant correlation between RDW and WBC in critically ill dogs. However, inflammation has also been postulated as a possible cause of increased RDW in dogs with multicentric lymphoma and bacterial pneumonia.

### 8.3. Oxidative Stress

Oxidative stress develops in many chronic diseases (e.g., cancer, cardiovascular disease, inflammation, liver failure, and chronic kidney disease) and is characterized by an imbalance between the formation of free radicals and the body’s antioxidant defenses. The resulting reactive oxygen species induce damage to macromolecules such as cellular proteins and lipids, resulting in cellular death and impaired vascular permeability [2,3,18]. Red blood cell membranes are extremely sensitive to these effects, resulting in impaired erythropoiesis, increased membrane fragility, and shortened RBC lifespan [61,64]. Some studies have shown a positive association between increased RDW and increased biomarkers of oxidative stress [5] and decreased plasma levels of natural antioxidants [57] in humans. These studies suggest that oxidative stress can cause anisocytosis. Similar studies are lacking in the veterinary literature. It has been postulated that the release of reactive oxygen species, which leads to a reduction in the half-life of circulating RBCs, was a possible trigger for the association between increased RDW and mortality in critically ill hospitalized dogs [20]. However, no experimental confirmation of this hypothesis has been given.

### 8.4. Deformability and Fragmentation Properties of RBCs

Red blood cells can carry oxygen and carbon dioxide in the blood due to their ability to deform and flow through microcapillaries, which are smaller in diameter than erythrocytes. This occurs due to the shape and size of RBCs and, above all, the deformability of their cell membrane. In humans, many pathological conditions can affect RBC deformability and elasticity [65,66,67]. No studies have yet demonstrated similar results in animal diseases.

Recent studies have shown that pro-inflammatory and oxidative states or nutritional deficiency also induce RBC fragmentation with release of microparticles into the blood [68]. In particular, RBC microparticles contain molecules capable of activating various processes, mainly negative, in other cells such as WBCs, platelets, and endothelial cells, leading to an increase in the pro-inflammatory, pro-thrombotic, and oxidative state, and anisocytosis. A close correlation has been found between increased RDW and increased blood levels of RBC microparticles in humans [69,70]. Microparticle studies are rare in dogs [71,72], and their correlation with RDW has not yet been evaluated.

## 9. Conclusions and Future Prospective

Recent evidence derived from several human studies have highlighted the clinical usefulness of RDW not only in patients with anemia but also in those affected by disorders of various organs or systems. This quick, low-cost, and easy-to-perform parameter can provide both diagnostic and prognostic information, and an increased in RDW is often associated with a poor outcome in many pathological conditions. In contrast, the results of studies conducted in dogs have shown conflicting results regarding the role of RDW in animals with different disorders. These studies were characterized by heterogeneous aims, study designs that were most often retrospective, and the type of canine disorders. Nevertheless, some recent studies reported the negative prognostic role of RDW in dogs with MVD, AP, and those admitted to ICU.

The number and type of animal disorders in which RDW has been evaluated are quite small compared to those reported in human patients. For example, no studies have explored the role of this parameter in small animals with cancer, or severe inflammatory state (e.g., peritonitis, pleuritis, systemic inflammatory response syndrome, and pyometra,). Furthermore, the existing veterinary literature is often focused on the retrospective comparison of the RDW value between sick and healthy animals, with limited evaluation of the prognostic role of this parameter. To this aim, monitoring the dynamic changes of RDW over time in the course of different diseases will provide further useful and more precise information on mortality, as already demonstrated in humans. Another limitation of the existing veterinary literature on the prognostic role of RDW is associated with the combined evaluation of this parameter in sometimes small groups of animals affected by different disorders (e.g., those hospitalized or admitted to an ICU and suffering from different and disparate diseases). More studies with larger groups of animals that focus on a single disease/disorder will allow for a more accurate assessment of the ability of increased RDW to predict adverse outcome in affected animals.

## Figures and Tables

**Table 1:** Summary of studies evaluating the role of RDW in different canine disorders.

Author, Year	Study Design	Study Population	Major Findings	References
Neiger et al., 2002	Retrospective cohort	74 dogs without hematological abnormalities, 51 dogs with regenerative anemia, and 92 dogs with non-regenerative anemia	For each 1% RDW increase, there could be an increase in the odds by a factor of 1.3 that an anemic dog can be affected by regenerative anemia	[1]
Guglielmini et al., 2013	Retrospective case-control study	27 healthy dogs and 135 dogs with mitral valve disease (MVD) with or without heart failure (HF)	Mean RDW in dogs with MVD was not significantly different from that of healthy dogs. The RDW of dogs with MVD without HF was not significantly different from that of dogs with MVD and HF	[10]
Swann et al., 2014	Retrospective case-control study	79 healthy dogs and 44 dogs with pre-capillary pulmonary hypertension (PH)	Median RDW was significantly increased in dogs with pre-capillary PH compared to that of dogs of the control group	[12]
Mazzotta et al., 2016	Prospective study	19 healthy dogs, 82 dogs with MVD with or without PH, and 26 dogs with pre-capillary PH	Median RDW in dogs with pre-capillary PH and post-capillary PH was significantly higher compared to that of control dogs. Positive association	[11]
Fish et al., 2019	Retrospective observational study	129 dogs with acute traumatic injury	RDW was not associated with survival in dogs with traumatic injury	[22]
Martinez et al., 2019	Retrospective study	79 healthy dogs and 276 dogs with various disorders including endocrine, neurological, respiratory, hematological, cardiovascular, hepatic, and pancreatic disease	RDW of dogs with immune-mediated hemolytic anemia, immune-mediated thrombocytopenia, hyperadrenocorticism, hypothyroidism, hepatic vascular anomaly, pneumonia, chronic kidney disease, multi-centric lymphoma, and MVD was significantly higher compared to that of healthy dogs	[18]
Kim et al., 2019	Prospective study	20 healthy dogs and 86 dogs with heartworm disease (HWD) at different stage of disease severity	Higher RDW values were found in dogs with severe HWD compared to that of the control group. RDW was significantly correlated with presence of PH and anemia	[17]
Ludwik et al., 2021	Retrospectivesingle-center study	5183 dogs admitted to ICU	Higher RDW values on presentation to ICU were significantly associated with greater odds of all-cause in-hospital mortality compared to dogs with lower RDW values (OR = 2.1, 95% CI, 1.7–2.6)	[20]
Garcia-Arce et al., 2022	Retrospective study	127 critically ill dogs admitted to ICU	RDW was not associated with in-hospital mortality or length of hospitalization	[19]
Pfeifer et al., 2022	Prospective observational study	111 dogs admitted to intensive care unit (ICU) with different illness severity	RDW was not associated with illness severity nor did it predict in-hospital or 30-day mortality	[21]
Guglielmini et al., 2021	Retrospective cohort study	146 dogs with MVD at different stages of disease severity	RDW was independently associated with all-cause mortality at six months (HR: 1.203; 95% CI: 1.045–1.384, *p* = 0.010)	[13]
Guglielmini et al., 2022	Retrospective cohort study	70 dogs with acute pancreatitis	Increased RDW was an independent predictor of dead within 14 days (HR: 5.08; 95% CI: 1.14–22.67, *p* = 0.03) and showed good accuracy to predict negative outcome (AUC: 0.74; 95% CI: 0.63–0.84)	[16]

## Data Availability

No new data were created or analyzed in this study. Data sharing is not applicable to this article.
